# A One Health Perspective on Cancer: A Narrative Review

**DOI:** 10.3390/medsci14020221

**Published:** 2026-04-29

**Authors:** Sílvia A. C. Duarte, Rosário Pinto-Leite, Felisbina L. Queiroga

**Affiliations:** 1 Department of Medical Oncology, Local Health Unit Trás-os-Montes and Alto Douro, 5000-508 Vila Real, Portugal; sicduarte@gmail.com; 2 CECAV, School of Agrarian and Veterinary Sciences, University of Trás-os-Montes and Alto Douro (UTAD), 5000-801 Vila Real, Portugal; 3 Genetics Laboratory, Local Health Unit Trás-os-Montes and Alto Douro, 5000-508 Vila Real, Portugal; mlleite@chtmad.min-saude.pt

**Keywords:** one health, cancer prevention, environmental risk factors, comparative oncology, microbiome, public health

## Abstract

Cancer is a major public health challenge worldwide, with increasing incidence and a growing economic and societal burden. Despite therapeutic advances, prevention remains the most effective strategy to reduce its impact. The One Health approach, which recognizes the interconnection between human, animal, and environmental health, provides a valuable framework to address cancer risk factors in a more integrated and sustainable way. This narrative review addresses cancer through a One Health lens. Human health aspects include the global burden, major lifestyle and infectious risk factors, and key prevention strategies. Environmental determinants of cancer are summarized with emphasis on climate change, air pollution, occupational exposures, microplastics, ultraviolet radiation, and nutrition/food safety. Animal health contributions include insights from comparative oncology, which offer translational opportunities for prevention, diagnosis, and treatment, and from microbiome research revealing promising biomarkers for early detection and treatment response. Integrating cancer prevention into the One Health framework is essential for addressing the complex interplay between environmental, animal, and human health. A multidisciplinary approach can enhance public health policies, promote sustainable prevention measures, and improve early detection and treatment strategies, ultimately reducing healthcare costs and advancing global health outcomes.

## 1. Introduction

One Health is an integrated approach that seeks to balance and optimize human, animal and environmental health, recognizing their close interdependence. Its implementation is grounded in principles of equity between disciplines, sociopolitical and multicultural parity, socioecological balance, and transdisciplinary collaboration [1]. This approach aims not only to limit the impact of global health crises but, more importantly, to prevent them [2]. While traditionally applied to zoonoses, antimicrobial resistance and environmental hazards, One Health relevance extends to chronic diseases, including cancer, whose determinants span biological, behavioral, and ecological domains.

Stressful variations in ecosystems caused by anthropogenic activities often trigger slow adaptive responses as systems strive to establish a new equilibrium [3]. Human-induced environmental changes, such as habitat degradation, deforestation, intensive agriculture, pollution, and climate change, disrupt ecosystems with direct implication for human well-being, through food security, biodiversity loss, emerging infections, and chronic diseases such as cancer [4,5]. These disruptions alter exposure to carcinogens, affect immune and metabolic health, and shape behavioral risk patterns. Integrating cancer into the One Health framework offers opportunities to reduce exposure to shared risk factors across species [6,7,8], encourage human behavioral changes to promote healthier lifestyles [9,10], foster translational research [11,12] and develop early detection mechanisms based on animal sentinel data [13] and ecosystem monitoring [14,15].

The One Health cross-disciplinary approach extends beyond biology, ecology and medicine to incorporate economics and social sciences. This broader perspective is particularly important given the economic burden of cancer, which poses a significant threat to the sustainability of healthcare systems, disproportionately affecting lower-income countries [16]. The rise in cancer-related health expenditures exceeds the increase in cancer incidence, a financial challenge projected to worsen in the coming years [17]. From a One Health perspective, strategies focusing on cost-effective prevention and equitable access to affordable care are essential to mitigate economic strain on vulnerable healthcare systems.

The purpose of this narrative review is to highlight the relevance of integrating cancer prevention and control within the holistic One Health framework to promote sustainable health practices. From a human health perspective, this review examines the global burden of cancer, considering its epidemiological, societal, and economic impacts. It explores key lifestyle risk factors and the oncogenic role of infections, as well as strategies for effective cancer prevention and control. From an environmental health standpoint, the review addresses critical links between cancer and factors such as climate change, air pollution, occupational exposures, microplastics, ultraviolet radiation, and issues related to nutrition and food safety. From an animal health perspective, it analyses the translational insights gained from comparative oncology and the influence of microbiomes on cancer.

## 2. Materials and Methods

This narrative review was developed through a structured literature search in the PubMed, Scopus, and Web of Science databases. No strict temporal restrictions were applied, however, priority was given to studies published within the past decade to ensure the inclusion of the most current evidence, while key earlier publications were included for contextual relevance. In addition, authoritative reports and online resources from international health organizations and professional societies, including the World Health Organization, International Agency for Research on Cancer, European Society for Medical Oncology, American Cancer Society, and the Food and Agriculture Organization of the United Nations, were consulted to incorporate current clinical guidelines and policy-relevant information. The literature search was conducted between January 2024 and December 2025.

The search strategy was adapted to each database using combinations of free-text terms and controlled vocabulary (MeSH in PubMed), with equivalent terms applied across platforms. No language restrictions were applied. Search strings combined core concepts related to cancer and One Health with environmental, animal, and epidemiological domains using Boolean operators (“AND”, “OR”). The following keywords were included: “cancer”, “One Health”, “cancer burden”, “cancer prevention”, “cancer control”, “environmental exposures”, “climate change”, “air pollution”, “occupational carcinogens”, “microplastics”, “ultraviolet radiation”, “food safety”, “comparative oncology”, and “microbiome”. Additionally, reference lists of selected articles were screened to identify additional relevant studies.

Eligible publications included peer-reviewed original experimental and observational research articles, narrative and systematic reviews, meta-analyses, consensus statements, and clinical guidelines that contributed to the understanding of cancer within a One Health perspective. Study selection was performed through screening of titles and abstracts followed by full-text assessment. Exclusion criteria included non-peer-reviewed sources, studies not directly relevant to the One Health framework, and publications lacking sufficient methodological detail or scientific rigor. Evidence was prioritized according to study design, with systematic reviews and meta-analyses considered higher-level evidence, followed by observational studies and experimental models. When human data were limited, findings from animal and in vitro studies were included to illustrate potential biological mechanisms. Conflicting findings were addressed through comparative evaluation of study quality, consistency, and plausibility.

Given the narrative nature of this review, a formal systematic review protocol was not applied, as the objective was to provide a broad, integrative, and interdisciplinary synthesis rather than a quantitative systematic analysis. Consequently, inherent limitations include potential selection bias, heterogeneity of sources, and the absence of formal systematic screening and quantitative synthesis. These limitations were considered in the interpretation of the findings.

## 3. One Health: Historical Context and Concept

The One Health underlying ideology has been recognized since the 19th century, with Rudolf Virchow (1821–1902) establishing comparative medicine by connecting disease processes in animals and humans [18]. The term “One Medicine” was introduced in 1976 by Calvin Schwabe, the pioneer of veterinary epidemiology, advocating for an integrated approach to human, animal, and environmental health.

While the concept of One Health is longstanding, it was only in the 21st century that it was formally established as a comprehensive health approach. A key milestone was the 2004 symposium in New York City, One World, One Health: Building Interdisciplinary Bridges to Health in a Globalized World, where experts from human and animal health fields identified 12 key priorities, known as the “Manhattan Principles”, aimed to prevent major diseases of the 21st century, ensuring the preservation of Earth’s biological integrity for future generations.

In 2008, the Food and Agriculture Organization of the United Nations (FAO), World Organization for Animal Health (WOAH), World Health Organization (WHO), United Nations International Children’s Emergency Fund (UNICEF), United Nations System Influenza Coordination (UNSIC) and The World Bank published a joint strategy titled Contributing to One World, One Health^™^, which emphasized emerging infectious diseases at the human–animal–ecosystem interface [19]. That same year, the International Ministerial Conference on Avian and Pandemic Influenza in Egypt, with participation from over 120 countries and 26 organizations, officially endorsed One Health as a global, political framework for addressing infectious threats such as H5N1.

In May 2010, the Centers for Disease Control and Prevention (CDC), WOAH, FAO, and WHO convened the Stone Mountain Meeting in Georgia to define actionable steps for implementing One Health. This was followed by the first International One Health Congress in Melbourne in 2011, which gathered participants from 60 countries to explore the benefits of a collaborative One Health approach.

Acknowledging the urgency of addressing future threats like the COVID-19 pandemic, the Quadripartite Organizations (WHO, FAO, WOAH, and UNEP) established the One Health High-Level Expert Panel (OHHLEP) in May 2021, an independent advisory group, to provide evidence-based scientific and policy recommendations on One Health-related matters that support improved cooperation among governments [2].

OHHLEP formally defined One Health as an integrated approach aimed at sustainably balancing and optimizing the health of humans, animals, and ecosystems. This definition emphasizes their interconnectedness and interdependence, highlighting the need for cross-disciplinary collaboration to enhance well-being, address public health challenges, and promote sustainable development [1]. To support this, OHHLEP developed the One Health Joint Plan of Action, which includes a Theory of Change and serves as a strategic framework for the Quadripartite and other aligned institutions [20].

During its first term (2021–2023), OHHLEP focused on preventing and mitigating zoonotic pandemics, as detailed in its 2023 Annual Report [21]. In its second term (2024-ongoing), the panel aims to broaden its scope across all One Health action areas and strengthen ties with regional and international initiatives.

A critical next step is expanding One Health’s focus to include noncommunicable diseases with high global impact, such as cancer, which are potentially preventable, strongly linked to environmental risk factors, and sometimes directly related to animal species. This broader application aligns with the principles of One Health and enhances its relevance for addressing 21st-century health challenges.

## 4. Global Burden of Cancer

Cancer is a leading cause of death globally and a significant contributor to the burden of noncommunicable diseases. Its societal impact is expected to grow as prevalence and morbidity increase, placing greater demands on cancer care services [22]. In 2022, an estimated 20 million new cancer cases were diagnosed, a number projected to increase to 35 million by 2050, with 19 million cases in males and 16 million in females, representing a 77% increase in less than 30 years [23]. Cancer-related deaths are projected to reach 18.5 million by 2050, reflecting an increase of approximately 50% [23].

When assessing the global landscape of cancer distribution, relevant aspects differentiate low- or middle-income countries from high- or very high-income countries. Table 1 presents global cancer statistics stratified by the Human Development Index (HDI) levels—very high, high, medium, and low—as defined by the United Nations Development Program (UNDP). The data were retrieved from the Global Cancer Observatory, developed by the International Agency for Research on Cancer (IARC) [24,25,26]. The most common cancer types are listed in order of frequency and the main risk factors associated with cancer burden are presented by HDI group.

**Figure 1:**
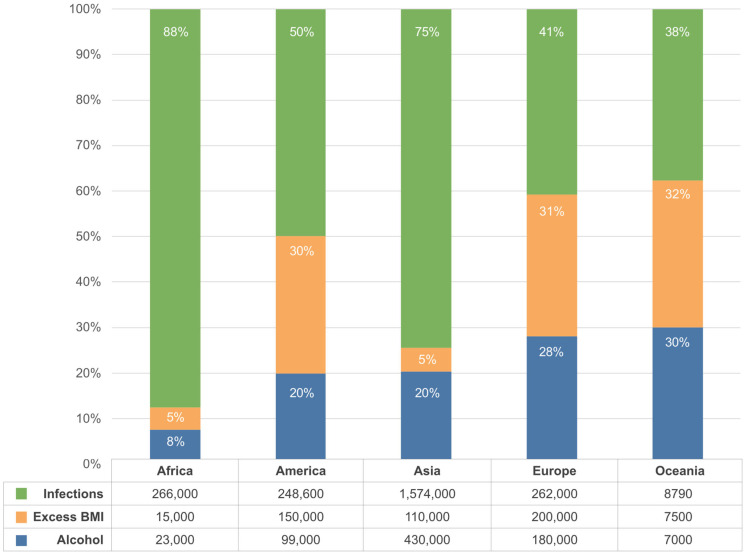
Estimated number of new cancer cases in 2020 attributable to alcohol consumption, excess BMI, and infections, by continent. The stacked columns represent the relative proportion (%) of cancer cases attributable to each risk factor within each continent, while the table reports absolute case numbers. Data source: Global Cancer Observatory, International Agency for Research on Cancer (IARC).

In low- or middle-income countries, infection-related cancers account for a substantial share of the burden, particularly cervical, liver, and gastric cancers linked to human papillomavirus (HPV), hepatitis B and C virus, and *Helicobacter pylori* [33]. Parasitic infections also play a role, with *Schistosoma haematobium* associated with bladder cancer and liver flukes (*Opisthorchis viverrini* and *Clonorchis sinensis*) with cholangiocarcinoma [31]. Globally, infectious agents are estimated to cause about 15% of all new cancer cases annually, with two-thirds occurring in low- and middle-income settings [32]. Cancers of the cervix, liver and stomach accounted for 2.5 million new cases globally in 2022, representing one in eight diagnoses and nearly one in five cancer deaths [23]. In contrast, high-income countries show a predominance of cancers associated with western lifestyle factors—such as lung, female breast, colorectal, and prostate cancer [33]—driven by obesity, physical inactivity, diet, smoking, alcohol, and reproductive patterns [27,28,29]. Figure 1 illustrates the estimated number of new cancer cases in 2020 attributable to alcohol consumption, excess high body mass index (BMI) and infections, stratified by continent [36,37,38].

However, in the last decade, low- and middle-income countries are experiencing an epidemiological transition with a change in the profile of the most common cancer types [33]. As the socioeconomic conditions and developmental status of these countries improve, there is a shift from a predominance of infection-related cancers to an increasing burden of cancers associated with western lifestyle risk factors and population aging [30,34]. In 2019, nearly half of global cancer deaths were attributed to modifiable risk factors, with smoking, alcohol consumption and high BMI being the leading contributors, while unsafe sex predominated in countries with low socio-demographic index [30]. Importantly, tobacco use remains the single most preventable cause of death and disease, and a major driver of global health inequities [35].

Within this context, particular attention is given to lung cancer in men and breast cancer in women, the most frequently diagnosed cancers worldwide. Lung cancer remains the leading cause of cancer mortality, with 2.2 million new cases and 1.8 million deaths in 2020 [39], predominantly driven by tobacco use, which accounted for 2.5 million cancer deaths globally in 2019 [40]. While smoking prevalence and lung cancer rates are declining in high-income countries, they are rising in many middle-income countries and remain high in Asia and parts of Europe, reflecting the delayed implementation of effective tobacco control measures [40,41,42]. Breast cancer became the most frequently diagnosed cancer globally in 2020, with 2.3 million new cases and 685,000 deaths, showing an 88% higher incidence in high-income countries due to the prevalence of reproductive, hormonal, and lifestyle risk factors, as well as increased detection through screening programs [39]. However, mortality is 17% higher in low- and middle-income countries, reflecting inequities in healthcare access and early detection [39]. The rising incidence in transitioning regions, such as South America, Africa, and Asia, reflects shifts in lifestyle and sociocultural norms, marked by women’s increasing participation in the workforce and greater access to birth control [41]. Additionally, increased life expectancy, obesity, and westernized dietary habits are further amplifying breast cancer risk. These evolving trends highlight the importance of integrating breast cancer prevention and control into broader public health strategies that consider social, cultural, and economic contexts [43].

Cancer is a leading cause of premature mortality [44]. In 2020, an estimated 183 million years of life lost (YLLs) were attributed to premature cancer death, of which two-thirds considered preventable, and one-third considered treatable. Lung cancer was the leading contributor to preventable premature YLLs in medium- and high-income countries, whereas cervical cancer led in low-income countries. Colorectal and breast cancers were the major treatable cancers across all countries, underscoring the substantial potential for reducing cancer mortality through both preventative measures and curative interventions [45].

Global cancer incidence and mortality patterns are heterogeneous and strongly influenced by levels of human development. Populations transitioning from lower to higher development levels show a decline in infection-related cancers, offset by a rise in cancers associated with westernized lifestyles and the tobacco epidemic [39]. Although the absolute burden is expected to remain higher in high-income countries due to aging populations, the fastest growth in incidence is predicted in low- and middle-income countries, with projected increases in new cases of 142% and 99% by 2050, respectively [23]. Without effective control strategies that prioritize both prevention and treatment, these increases could severely strain healthcare systems in economically fragile regions already challenged by limited resources and humanitarian crises [46]. Importantly, these comparisons are based on population-level data and should not be interpreted as reflecting individual-level risk or causal relationships. Differences observed across development levels may be influenced by multiple contextual factors, including healthcare access, diagnostic capacity, demographic structure, and exposure distributions.

The escalating global cancer burden is driven by population growth and aging, alongside shifts in exposure to risk factors often linked to socioeconomic development. Globally, the burden of cancer is significant in every country, regardless of development level, and is expected to continue rising in the future.

## 5. Economic Burden of Cancer

Cancer management is often linked to substantial financial burdens. These arise from direct costs, including expenses for diagnostics, treatments, and hospitalizations, as well as indirect costs from lost productivity due to morbidity and premature mortality, collectively exerting significant economic pressure [17,47,48,49]. The rising costs of innovative treatments combined with improved survival and an aging population further amplify the financial toxicity of cancer care [50].

**Figure 2:**
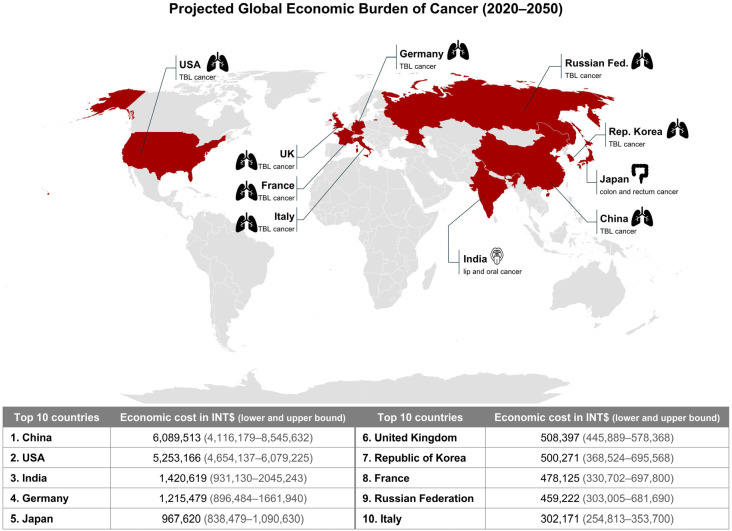
Projected Global Economic Burden of Cancer (2020–2050). Map illustrates the total projected cancer-attributable macroeconomic cost by country, measured in INT$ and discounted at an annual rate of 2%. The top five countries bearing the highest economic burden are highlighted and labeled with their leading contributor cancer type by cost. Data source: Chen et al., JAMA Oncol. 2023; 9(4): 465–472 [16]. INT $—international dollars at constant 2017 prices; USA—United States of America; TBL—tracheal, bronchus, and lung; UK—United Kingdom.

The global economic burden of cancer from 2020 to 2050 was estimated to be INT $25.2 trillion. China bears the highest economic cost of cancer, estimated at INT $6.1 trillion, followed by the United States of America at INT $5.3 trillion and India at INT $1.4 trillion (Figure 2). The three cancers with the highest economic burden were lung cancer (INT $3.9 trillion), colorectal cancer (INT $2.8 trillion) and breast cancer (INT $2.0 trillion) [16]. However, such estimates are influenced by healthcare system capacity, expenditure patterns, and modelling assumptions, and should therefore be interpreted cautiously rather than as direct measures of prevention needs or opportunities.

The economic burden of cancer varies, not only by cancer type but also across income groups. High-income countries faced an economic loss of INT $12.8 trillion, equivalent to INT $10,294 per capita. In contrast, lower-income countries incurred a substantially smaller burden of INT $168 billion, translating to a per capita loss of INT $174 [16]. While high-income countries incur the greatest macroeconomic costs, low- and middle-income countries bear most of the human burden, compounded by the lack of infrastructure, inadequate funding, and limited access to affordable treatments [51]. Particularly in low-income countries, investing in cancer care delivers substantial health and economic benefits [52].

Individually, cancer patients often face financial challenges due to direct medical and non-medical costs associated with diagnosis, treatment, and follow-up [53], as well as indirect costs from reduced productivity or job loss [48,54]. They are 71% more likely than the general population to experience a severe adverse financial event [55]. This phenomenon, known as financial toxicity, negatively affects both survival outcomes [56] and quality of life [57], as it encompasses multiple domains: the psychological domain, contributing to anxiety, depression, and fatigue [58]; the behavioral domain, leading to reduced adherence to treatment due to high costs [59]; and the material domain, manifesting as housing and food insecurity [60]. With the rising costs of cancer care, it is imperative for clinicians, oncology organizations, and health systems to collaborate in raising awareness and alleviating the financial burden on patients.

Tobacco, the leading preventable cause of cancer worldwide [61], represented, in 2012, a total economic cost, including healthcare expenses and productivity losses, estimated at US $1436 billion [62]. It is estimated that if China, the world’s largest producer and consumer of tobacco, implemented a comprehensive tobacco control measures it could save US $1 trillion between 2015 and 2030, accounting for the preventable burden of non-communicable diseases (NCDs) attributable to tobacco use, including cancer [63].

Investing in prevention guided by One Health principles is projected to significantly reduce the costs associated with crisis response [64]. Prioritizing effective health interventions to mitigate the cancer burden is essential to ensuring economic stability.

## 6. Prevent and Control Cancer

To curb the anticipated rise in cancer incidence and costs, it is essential to prioritize strategies that promote access to comprehensive and cost-effective cancer prevention, early diagnosis, treatment, and survivorship and palliative care.

Cancer control is a growing global health priority, prompting international organizations to set measurable goals and implement strategies to reduce incidence, improve outcomes, and address care disparities. In May 2017, governments reaffirmed this commitment by adopting World Health Assembly Cancer Resolution 70.12. The WHO’s Global Action Plan for the Prevention and Control of NCDs 2013–2030 set a target of reducing premature mortality from NCDs, including cancer, by 33.3% by 2030. The European Commission developed the Europe’s Beating Cancer Plan supports Member States to address the cancer care continuum through research and innovation. At the 77th World Health Assembly in June 2024, priorities were reiterated: strengthening health systems; addressing modifiable risk factors (regulation and taxation particularly of tobacco, alcohol, and unhealthy foods); tackling social, environmental, and commercial determinants of cancer; addressing the cancer workforce crisis; accelerating cervical cancer elimination; reducing financial burdens; and investing in research, innovative, and digital health [65].

Prevention, encompassing its five levels, is an effective strategy for cancer control, with up to half of all cancers being preventable [66]. Although such estimates are useful to illustrate the potential preventable burden of cancer, they should be interpreted cautiously, as results may vary according to expo-sure prevalence, modelling assumptions, causal frameworks, counterfactual scenarios, and source populations across studies. Primordial prevention addresses the underlying social, environmental, and economic determinants of health to reduce risk factors. Primary prevention lowers risk through lifestyle modifications, elimination of carcinogenic exposures, like tobacco, alcohol and air pollution, health promotion strategies and proactive health measures such as healthy eating, exercise, vaccination, risk-reducing surgeries, and chemoprevention. Secondary prevention enables early detection through screening and treatment of precancerous lesions. Tertiary prevention improves quality of life by minimizing treatment toxicities, reducing disability and enhancing survivorship or palliative care. Quaternary prevention protects against unnecessary interventions, avoiding harm from overdiagnosis or overtreatment of low-risk conditions [67].

**Tobacco** smoking is a major preventable contributor to cancer morbidity and premature mortality [68]. Classified by the International Agency for Research on Cancer (IARC) as carcinogen, it is linked to multiple cancers, including cancers of the head and neck, lung, esophagus, stomach, colorectum, pancreas, uterine cervix, kidney, renal pelvis, ureter, and urinary bladder, chronic myeloid leukemia and acute myeloid leukemia [69]. Countries implementing strong tobacco control measures, particularly under the WHO Framework Convention on Tobacco Control, have achieved marked declines in smoking prevalence. Effective strategies include higher taxation, smoke-free legislation, public education, advertising bans, health warnings or standardized packaging, and access to cessation and rehabilitation treatments. Strengthening these measures could prevent approximately 1.65 million lung cancer cases over 20 years, representing 19.8% of projected new lung cancer cases in men and 23.2% in women [70]. Among modifiable risk factors, tobacco cessation offers the greatest potential for cancer prevention, capable of averting millions of deaths annually.

**Alcohol** has been classified as a carcinogen by the IARC since 1988, with sufficient evidence linking its consumption to hepatocellular carcinoma, esophageal squamous cell carcinoma, oral cavity, pharynx, larynx, colorectal, and breast cancer [69]. Globally, alcohol consumption is associated with approximately 740,000 new cancer cases annually [71]. In the European Union, light to moderate alcohol consumption caused nearly 23,000 new cancer cases in 2017, representing 13.3% of all alcohol-related cancers, with almost half occurring in women with breast cancer [72]. While traditionally more common in men, alcohol consumption, particularly binge drinking, is rising among women [73]. There is no evidence that alcohol’s purported cardiovascular benefits mitigate cancer risk, nor is there a safe threshold of consumption below which the carcinogenic effects of alcohol are absent [74]. Complementing efforts to enhance health literacy, the WHO’s Less Alcohol Unit focuses on addressing the underlying factors that influence the acceptability, availability, and affordability of alcohol. These efforts aim to accelerate the adoption of high-impact interventions as part of the Global Strategy to Reduce the Harmful Use of Alcohol.

Cervical cancer illustrates the effectiveness of integrated prevention. HPV **infection** causes 99% of cases [75], making it highly preventable through primordial prevention (promote health literacy encouraging safe sex practices), primary prevention (HPV vaccination), secondary prevention (population-based screening programs and treatment of precancerous lesions), and tertiary prevention (treatment of early-stage disease). The Europe’s Beating Cancer Plan targets 90% HPV vaccination coverage for girls and increased coverage in boys by 2030. The WHO aims to eliminate cervical cancer as a public health problem by reducing incidence to below 4 per 100,000 women [76]. However, prevention is unevenly implemented, with low-income countries bearing a disproportionate burden, where the incidence and mortality rates exceed the threshold set by the WHO [77]. Beyond HPV, approximately 90% of other infection-related cancers worldwide are attributed to *Helicobacter pylori*, hepatitis B virus (HBV), and hepatitis C virus (HCV), all preventable or treatable through vaccination, treatment and behavioral interventions that limit their transmission [78].

Addressing the **commercial determinants of health** is essential for cancer prevention. A WHO Regional Office for Europe report shows that harmful products, (including tobacco, alcohol, ultra-processed foods, sugar-sweetened beverages, and fossil fuels) and associated industry practices contribute significantly to rising cancer and NCD rates. These factors cause approximately 2.7 million deaths annually, equivalent to over 7400 daily deaths, accounting for nearly 25% of all deaths in the WHO European Region. The report calls for strong financial reforms and strict regulation to curb industry influence [79].

While several modifiable cancer risk factors are well-documented, advancing prevention requires stronger political commitment. Improving health literacy, especially among young people, is pivotal in promoting behavioral changes that reduce cancer risk, including minimizing tobacco and alcohol use, maintaining a healthy lifestyle through diet and exercise, practicing safe sex, protecting against sun exposure, and participating in routine cancer screening programs [80].

The 2021 NCD Country Capacity Survey assessed global progress in NCD prevention and control. Taxation on alcohol and tobacco was reported by 97% and 88% of countries, respectively. National screening programs for breast and cervical cancer were reported by 63% and 69% of countries, respectively, but were far more common in high-income settings. Access to cancer diagnosis and treatment improved modestly, yet inequalities remained. Radiotherapy was accessible in only 26% of low-income countries compared to 89% of high-income countries, while cancer centers or specialized departments were present in 56% and 93% of these countries, respectively. The availability of palliative care was globally limited, with community- or home-based care reported by 43% of countries [81]. A WHO survey revealed significant gaps in cancer care coverage, with only 39% of countries providing essential cancer management within universal health coverage and just 28% including palliative care [51]. These disparities drive excess morbidity, mortality, and financial hardship, underscoring the urgent need for increased investment in comprehensive cancer services.

While addressing modifiable cancer risk factors is essential for fostering a healthier society, it will not eliminate the cancer burden, since incidence is closely linked to aging. Projections suggest that even with a hypothetical total elimination of smoking, obesity, and alcohol consumption, the incidence of breast and colorectal cancers would remain unchanged by 2050 compared to 2021. This outcome reflects the overwhelming influence of demographic shift, which surpasses the potential of prevention efforts [82]. As population aging drives cancer incidence, control strategies must extend beyond risk reduction to include early diagnosis and effective, accessible, and affordable treatments for all patients. Additionally, in ageing populations, competing risks of death may attenuate the observable impact of preventive interventions on absolute cancer risk, highlighting the need for nuanced interpretation of prevention benefits.

Clinicians, as independent stakeholders in the healthcare system, play a role in advocating for optimal patient outcomes and ensuring that cancer control strategies reflect local realities, including prevalent cancer types and available resources. The European Society for Medical Oncology (ESMO) Leaders Generation Program has outlined national-level priorities for policymakers, health planners, clinicians, patients, and civil society to improve global access to high-quality cancer care [83]. In preparation for the 2025 UN High-level Meeting on NCD prevention and control, ESMO issued further recommendations to accelerate cancer prevention and equitable care. Key actions include: promoting universal health coverage to ensure equitable access without imposing financial hardship on patients; comprise cancer services into health emergency and pandemic preparedness; strengthening the oncology workforce through education, training and retention; enhancing diversity and inclusivity in clinical trials; securing public funding for independent research; and advancing prevention through stricter tobacco and alcohol control, expanded HPV and HBV vaccination, and air quality legislation aligning with the WHO recommendations to lower the annual limit value for fine particulate matter (PM_2.5_) to 5 μg/m^3^.

Research investment is essential for driving innovation in oncology. While clinical trials are widely acknowledged as a pivotal tool for advancing patient care and expediting the development and commercialization of groundbreaking treatments, patient enrolment remains below 5% [84]. In November 2024, EU Member States endorsed the Budapest Declaration on the New European Competitiveness Deal, committing to allocate 3% of GDP to research and development by 2030.

To effectively reduce the global burden of cancer, it is crucial to recognize that a substantial proportion of cancers are preventable and that prevention strategies are both impactful and cost-effective. Cancer control policies should therefore be tailored to regional needs and allocate balanced investments across prevention, treatment, and palliative care to maximize health outcomes and reduce cancer-related morbidity and mortality.

## 7. Cancer Through the One Health Lens

The One Health concept meets the challenges of modern medicine by providing a holistic approach to understand diseases arising from the complex connections among humans, animals, and the environment. A key challenge in operationalizing the One Health framework in oncology lies in the methodological integration of data across these three domains. Integrated exposure modelling approaches can combine environmental monitoring data with individual- and population-level health information to better characterize cumulative and interacting determinants of cancer risk. Complementing this, cross-species surveillance can identify shared cancer patterns and sentinel events in animal populations that may signal environmental hazards relevant to humans. Translational epidemiology further strengthens this framework by linking findings from experimental models and veterinary oncology with human studies, improving inference regarding carcinogenic mechanisms. Together, these integrative methodologies allows a more comprehensive understanding of cancer and support more informed policy design, enabling alignment of environmental regulation, occupational safety measures, and public health interventions based on converging evidence across systems [85].

### 7.1. Environmental Health and Cancer

**Figure 3:**
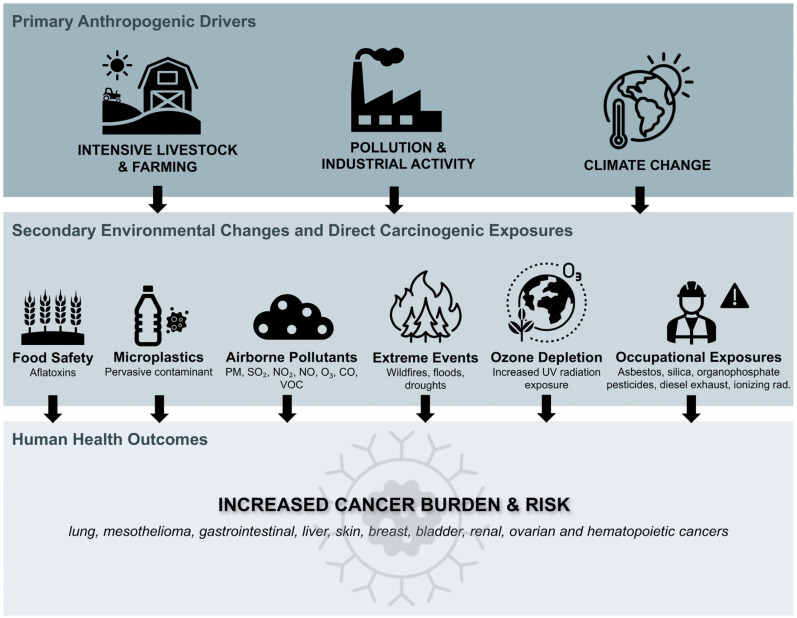
The Interconnected Pathways from Environmental Disruption to Human Cancer. Schematic representation of the pathways linking primary anthropogenic drivers to increased cancer risk through environmental mediators. The One Health perspective emphasizes that disruptions in environmental health are not isolated but are interconnected systems that directly and indirectly impact human health. Climate change and pollution act as force multipliers, exacerbating multiple exposure pathways simultaneously.

Cancer development is increasingly recognized as influenced by environmental factors. A One Health perspective underscores the interplay between environmental changes and cancer risk, revealing how disruptions to ecosystems and exposure to carcinogenic substances contribute to disease (Figure 3).

#### 7.1.1. Climate Change

The impact of climate change on climate-sensitive infectious diseases, food security, and undernutrition are well documented [86]; however, its effects on chronic diseases such as cancer remain less clear. Greenhouse gases released into the atmosphere by human activities are the primary drivers of global warming, causing a steady increase in the Earth’s surface temperature [87]. This leads to a cascade of environmental shifts such as sea-level rise, droughts, floods, wildfires, and extreme weather events, which indirectly affect cancer risk factors such as air pollution, ultraviolet radiation exposure, food and water insecurity, while also facilitating the spread of infections by carcinogenic agents. These effects are mainly linked to cancers of the lung, upper respiratory tract, skin, gastrointestinal tract, and liver [15]. The resulting strain on healthcare systems is expected to difficult access to health care and disrupt essential resources for effective cancer control [88]. Furthermore, cancer patients are a vulnerable population to the health threats of climate change due to the physical, psychological, and socioeconomic consequences of cancer diagnosis and treatment [89].

#### 7.1.2. Air Pollution

Outdoor air pollution is classified by the IARC as a Group 1 carcinogen. Household air pollution from burning of coal is also classified as a Group 1 carcinogen, and household burning of biomass fuel as Group 2A. Major ambient air pollutants, largely from fossil and biomass fuel combustion, include particulate matter (PM), sulfur dioxide, nitrogen dioxide, nitrogen oxide, carbon monoxide, ozone and volatile organic compounds [90]. PM encompasses a wide range of chemically and physically varied aerosols comprising solid particles or liquid droplets suspended in the air. Fine particulate matter (PM_2.5_), defined as airborne particles or droplets with an aerodynamic diameter ≤ 2.5 µm, is primarily originated from combustion and poses significant health risks due to its toxic compounds, such as heavy metals, and its ability to penetrate deep into the respiratory tract and interact with mucosal surfaces [91]. PM_2.5_ exposure patterns vary with sociodemographic development. Household air pollution is higher at lower social development index (SDI) and declines as sociodemographic conditions improve. In contrast, ambient PM rises with industrialization and subsequently declines as air quality management measures are implemented at higher SDI levels [30]. The Global Burden of Disease Study 2019 identified air pollution as one of the top five leading causes of attributable deaths worldwide [30]. Short-term exposure to PM_2.5_ and PM_10_ is linked to cardiovascular, respiratory, and cerebrovascular mortality [92]. Furthermore, several meta-analyses of cohort studies reported significant adverse associations between PM_2.5_ exposure and lung cancer incidence and mortality [90,93,94,95]. A mechanistic basis for PM_2.5_ driven lung cancer, particularly among never-smokers and in the absence of classical carcinogen-driven mutagenesis, has been proposed [96]. Additionally, air pollution exposure in lung cancer patients worsens clinical outcomes, increasing hospital admissions [97] and reducing survival in early stage non-small cell lung cancer [98]. Beyond lung cancer, systemic absorption of pollutants suggests possible associations with other cancers, including breast [99,100,101], bladder [102], brain [103], gastric [104], and hematopoietic cancers [105], though evidence remains less robust. The WHO’s 2021 air quality guideline recommends that annual average PM_2.5_ concentrations not exceed 5 µg/m^3^, yet most urban populations live in areas exceeding this threshold [106]. It is estimated that nearly one-third of PM_2.5_-attributable deaths between 2000 and 2019 could have been prevented if cities had met the previous WHO 2005 guideline of 10 μg/m^3^ [107]. Given the link between rising PM_2.5_ concentrations and cancer burden a comprehensive public health strategy is essential to both mitigate emissions and prevent pollution-related cancer.

#### 7.1.3. Occupational Exposures

Estimating the cancer burden attributed to occupational exposures is challenging due to exposure variability, long latency periods between exposure and cancer development, and the lack of comprehensive data, particularly in low- and middle-income countries [108]. From 1990 to 2017, asbestos, silica, and diesel engine exhaust were the three leading occupational carcinogens [109]. Certain associations between exposures and specific cancers are well-established, including asbestos with mesothelioma, lung, laryngeal, and ovarian cancers [110]; arsenic with bladder, kidney, skin, liver, and colon cancers [111], and ionizing radiation with multiple cancer types [112]. Pesticides are widely used, resulting in significant occupational and environmental exposure globally. Research conducted mostly in vitro and animal models demonstrated the carcinogenic potential of pesticides, revealing their capacity to induce genotoxicity, hormone disruption, oxidative stress, inflammation, and immune modulation [113,114]. In 2017, the IARC classified five organophosphate pesticides (tetrachlorvinphos, parathion, malathion, diazinon and glyphosate) as probably (Group 2A) or possibly carcinogenic to humans (Group 2B). Despite subsequent high-quality studies, evidence in human carcinogenicity remains incomplete, and numerous potentially carcinogenic pesticides were not included in the IARC assessment [115]. Identifying such chemicals is a public health priority to guide regulatory actions in order to reduce cancer incidence.

#### 7.1.4. Microplastics

The widespread distribution of microplastics, defined as plastic particles smaller than 5 mm, is well-documented, with their presence confirmed across all environmental compartments, including marine and freshwater ecosystems, soils, atmosphere, and biota [116], and entering the food chain [117], particularly through plastic food-contact materials [118]. Microplastics pose a threat not only to the environment, but also to human health. Human exposure occurs mainly via ingestion, inhalation, and dermal contact [119]. Once internalized, microplastics can damage cell membranes, impair detoxifying mechanisms, induce oxidative stress, inflammation, genotoxicity, metabolic disruption, and cell death. These effects are often interconnected, reflecting the complex interplay of microplastic-induced cytotoxicity [119,120,121]. Although the cytotoxic effects of microplastics are well-established, growing concerns about their carcinogenic potential have prompted active research. Experimental studies have identified several mechanisms that may be associated with carcinogenic processes, including: persistence and bioaccumulation within biological systems [122,123]; size-dependent reactive oxygen species generation and subsequent DNA damage [124]; epigenetic regulation through DNA methylation, histone modifications, and non-coding RNA regulation, which modulate gene expression patterns associated with cancer initiation and progression [125,126]; chronic inflammation and immune modulation that reshape the tumor microenvironment while impairing immune surveillance [127,128]; endocrine disruption with potential links to hormone-related cancers [129,130]; and enhance cell migration, potentially facilitating metastasis [123]. The presence of microplastics in tumor cells has also been associated with disease progression in breast cancer, ovarian cancer, gastric cancer, cutaneous squamous cell carcinoma [131,132,133,134,135] and with treatment resistance in gastric cancer [136]. Most evidence derives from in vitro on human cell lines or in vivo on animal models, with scarce human exposure data particularly among occupationally exposed populations [137]. Emerging observational data exploring proxy exposure pathways, such as bottled water consumption, suggest potential population-level exposure patterns associated with selected non-cancer health outcomes, although no causal relationship with cancer has been established to date [138]. Comprehensive risk assessment of microplastics and their potential impact on cancer remains incomplete, hindering timely decision-making for health policies and effective mitigation strategies.

#### 7.1.5. Ultraviolet Radiation

The cause–effect relationship between increased exposure to ultraviolet (UV) radiation and the rising incidence of squamous cell carcinoma, basal cell carcinoma, and melanoma is well-established [139]. Since 1992, the IARC has classified solar and UV radiation as Group 1 carcinogens. While skin cancer risk increases with age, cumulative lifetime UV exposure remains the main driver [140]. UV radiation promotes tumorigenesis through gene mutations, epigenetic alterations, and immunosuppression [141]. Ozone depletion, driven by anthropogenic atmospheric emissions of aerosols such as chlorofluorocarbons, has led to increased UV radiation exposure at the Earth’s surface, significantly contributing to the global burden of skin cancer [15]. In response to the urgent need to protect the stratospheric ozone layer, the 1987 Montreal Protocol on Substances That Deplete the Ozone Layer was enacted. Since its implementation, significant reductions in ozone-depleting substances have been observed [142], mitigating a projected increase of up to 2 million additional skin cancer cases by 2030 [143]. However, the persistence of long-lived compounds and the continued emissions of unregulated compounds like dichloromethane, delays ozone layer recovery until at least 2060, perpetuating the hazard posed by increased UV radiation for several decades [144]. Additionally, there is an interplay between climate change and ozone depletion that influences climate patterns and UV radiation intensity. For instance, reduced cloud cover and higher UV irradiance at lower latitudes are expected to exacerbate the incidence of skin cancer in these regions [145]. Beyond skin cancer, UV radiation and ozone depletion adversely affect ocular health, immune system function, and ecosystems, thereby threatening food security and exacerbating climate-related health challenges [144]. Moreover, rising temperatures linked to climate change alter human behavior, increasing sun exposure through outdoor activities and reduced use of protective clothing, which further contributes to higher skin cancer incidence [146].

#### 7.1.6. Nutrition and Food Safety

In relation to **nutrition**, a diet rich in whole grains, vegetables, fruit and beans is protective against cancer susceptibility, whereas consumption of red and processed meats, sugar-sweetened beverages, and alcohol, as well as body fatness and obesity resulting from unhealthy diet and physical inactivity, raises risk [147]. Concerns regarding health, food security, and environmental sustainability are increasingly associated with the production and consumption of red and processed meats. Over the past 50 years, global meat production and consumption have risen significantly and are projected to increase by an additional 50% by 2050 [148]. Intensive livestock farming contributes to unsustainable environmental consequences, including greenhouse gas emissions, excessive freshwater use, land degradation, and biodiversity loss [149]. While red meat provides essential nutrients, such as highly bioavailable iron and vitamin B12, excessive intake is linked to an elevated risk of NCDs, including cancer [149]. In 2015, IARC classified consumption of red meat as probably carcinogenic (Group 2A) and processed meat as carcinogenic to humans (Group 1), with strong evidence for colorectal cancer risk and growing evidence for breast, endometrial, esophageal squamous-cell carcinoma, lung, renal cell, and hepatocellular carcinomas, extending the range of risks initially reported by the IARC [150]. In the United States, a 30% reduction in processed meat consumption over a decade could prevent 53,300 cases of colorectal cancer and 16,700 deaths [151]. Strong evidence supports the protective effects of plant-based diets in reducing cancer incidence and mortality [152,153], as well as the harmful effects of high red and processed meat consumption. Dietary shifts limiting red and processed meats benefit both human health and environmental sustainability by reducing greenhouse gas emissions and improving food security particularly in vulnerable populations [154]. A modelling study estimated that by 2050, changes in food supply due to climate change, including reduced availability of fruits and vegetables, could result in approximately 534,000 global deaths, including deaths from cancer. Notably, the health impacts of dietary and weight-related factors linked to climate change may surpass other climate-related health challenges [155]. 

In the context of **food safety**, mycotoxins illustrate how food safety intersects with cancer risk. Produced by fungi such as *Aspergillus*, *Penicillium*, and *Fusarium*, they thrive under warm and humid conditions, often exacerbated by monsoons, unseasonal rainfall during harvest, and flash floods [156]. Major mycotoxins, including aflatoxins, ochratoxins, fumonisins, deoxynivalenol, and zearalenone, frequently co-occur in contaminated food, amplifying their toxic effects through synergistic interactions [157]. Contamination can occur at any point in the food supply chain, from pre-harvest to processing and storage. Consumption of mycotoxin-contaminated food poses serious health risks to humans and animals, including acute and chronic effects such as hepatotoxicity, nephrotoxicity, neurotoxicity, immunotoxicity, and carcinogenicity, thus highlighting their critical impact on public health and food security [156]. Aflatoxins, produced by *Aspergillus* species, have been classified by IARC as carcinogenic to humans (Group 1), primarily due to their strong association with hepatocellular carcinoma. The interaction between mycotoxins and oncogenic viruses in human carcinogenesis has been documented, with aflatoxin B1 consistently associated with hepatocellular carcinoma in individuals infected with HBV [158]. Emerging evidence indicates that aflatoxins are likely to become a significant concern in regions previously unaffected, including Europe [159]. Climate change, through rising temperatures and water scarcity, is expected to increase the risk of aflatoxin contamination in maize throughout Southern and Central Europe within the next three decades, driven by environmental conditions favorable to *Aspergillus flavus* proliferation and subsequent aflatoxin B1 production. Additionally, climate change-induced abiotic stress is anticipated to weaken the resilience of crops, thereby making them more susceptible to fungal diseases [160,161]. Prevention, continuous monitoring, and detoxification strategies are essential to safeguard food safety, limit cancer risks, and protect vulnerable economies [162].

### 7.2. Animal Health and Cancer

The One Health perspective highlights how comparative oncology offers valuable insights into tumor biology and how microbiome research reveals promising biomarkers for early detection and treatment response.

#### 7.2.1. Comparative Oncology

The biological complexity of cancer has driven the development of increasingly sophisticated experimental models. In vitro systems have evolved from simple 2D monolayer cell cultures and spheroid cultures to more advanced 3D organoid cultures and microfluidic models, such as tumor-on-a-chip platforms [163]. Similarly, in vivo models have progressed from patient cell line-derived xenografts in immunodeficient mice to patient-derived xenografts and genetically engineered mouse models to transgenic models in non-mouse species designed to develop tumors and naturally occurring tumors in companion animals [164].

Comparative oncology is a multidisciplinary field of research that studies naturally occurring cancers in animals, particularly companion animals, to gain insights into cancer biology, prevention, and treatment [165] (Table 2).

However, such estimates are influenced by healthcare system capacity, expenditure patterns, and modelling assumptions, and should therefore be interpreted cautiously rather than as direct measures of prevention needs or opportunities [171,176,177]. While murine models have been useful for studying cancer biology, they often fail to accurately reflect key characteristics of human cancers [178]. Pet dogs are among the most extensively studied models in comparative oncology research due to compelling factors: they exhibit a relatively high tumor incidence [179]; share significant histopathological characteristics with human cancers, including tumor heterogeneity and microenvironmental interactions, molecular and genetic features, such as tumorigenesis signaling pathways, oncogenic drivers, mutational profiles and epigenetic signatures [180,181]; demonstrate strong parallels in biological behavior, including patterns of response or resistance to conventional therapies, metastasis, and recurrence [11]; cancers occurring naturally in immunocompetent animal models experience complex interactions between the tumor and the immune system enabling the evaluation of immunotherapy targets and the assessment of efficacy [181]; and their cohabitation with humans exposes them to similar environmental risk factors, making them valuable sentinels for identifying potential environmental carcinogens [182,183].

Comparative oncology clinical trials are increasingly valued for their ability to rapidly evaluate drug safety and efficacy, leveraging the shorter lifespans of companion animals to accelerate drug evaluation, eliminating agents with poor therapeutic indices and prioritizing those likely to succeed in Phase I human trials. They also allow access to innovative treatment options for pets when conventional therapies are ineffective. These efforts ultimately advance both veterinary and human oncology [177,178]. A reverse-translational approach integrates naturally occurring animal cancers into preclinical research and applies human data to improve veterinary care, promoting efficient, bidirectional knowledge exchange [184].

The interdisciplinary field of comparative oncology bridges human and veterinary medicine, providing a unique platform to investigate carcinogenesis across species. By integrating scientific findings from both domains, researchers can uncover molecular and genetic profiles that lead to the identification of novel therapeutic targets and the development of innovative treatments, ultimately benefiting both humans and animals. This approach has already yielded insights into tumor biology and has accelerated the translation of research findings into clinical practice [165,177].

#### 7.2.2. Microbiome

The human microbiome refers to the collective genome of microorganisms, including bacteria, fungi, viruses, and archaea that colonize the human body. Its metagenome exceeds the human genome by approximately 100-fold, providing vast genetic and functional diversity [185]. Moreover, microbiome composition is highly individualized, varying not only between individuals but also within a single person over time, influenced by factors such as age, environment, and health status [186].

In healthy states, the microbiota engages in a symbiotic relationship with the host, contributing to physiological processes such as digestion, metabolism, and immune system regulation [187,188,189]. However, disruptions from infection [190], antibiotic overuse [191], or unhealthy lifestyle behaviors, including diets high in processed foods and low in fiber, physical inactivity and nicotine exposure [192], can cause microbial dysbiosis, an imbalance linked to chronic inflammation, immune evasion, and oncogenic metabolites, which collectively promote carcinogenesis [193]. Low-biomass microbial populations have been detected in previously considered sterile tissues, such as the pancreas and liver, highlighting their potential influence on cancer biology [193].

Certain microorganisms are recognized as key contributors to carcinogenesis: *Helicobacter pylori* is strongly linked to gastric cancer, primarily through its ability to induce chronic inflammation and disrupt cellular signaling; *Fusobacterium nucleatum*, pathogenic *Escherichia coli*, and enterotoxigenic *Bacteroides fragilis* are implicated in colorectal cancer; and *Salmonella enterica* has been associated with gallbladder cancer [193]. Dysbiosis has also been linked to the onset and progression of lung cancer [194], head and neck squamous cell carcinoma [195], and breast cancer [196]. Furthermore, the intratumoral microbiome, comprising distinct microbial ecosystems within tumor tissues that vary across cancer types [197], appears to be organized in microniches that interact with immune and epithelial cells that support cancer progression [198], and may influence treatment efficacy and prognosis [199,200]. However, the directionality and causality of these associations remain uncertain and may be influenced by confounding factors.

While dysbiosis may lead to drug resistance, a healthy microbiome can enhance treatment efficacy. Efforts to modulate the microbiome as a therapeutic strategy are actively being explored, including fecal microbiota transplantation, probiotics, prebiotics, antibiotics, phage therapy, and dietary interventions [193,201]. Microbial signatures are emerging as promising biomarkers for early cancer detection, prognosis, and treatment stratification in colorectal [202], lung [203], and breast cancers [204].

The field of cancer microbiome research holds immense potential for advancing diagnostics and therapeutics. Microbial signatures in tumors and body fluids could serve as biomarkers for early cancer detection, while microbiome-targeting therapies offer opportunities to optimize treatment efficacy. Despite these promising developments, significant challenges remain in clarifying the causal relationships between microbiota and cancer, emphasizing the need for further research to fully harness the potential of this emerging field [201].

## 8. Conclusions and Future Directions

The global burden of cancer is escalating driven by demographic changes, persistent exposure to modifiable risk factors, and widening disparities in prevention and care. This growing burden places substantial pressure on health systems, economies, and societies worldwide. A One Health perspective provides a comprehensive framework to address the multifactorial nature of cancer, recognizing the interconnected influences of human behavior, environmental change, microbial ecosystems, and cross-species insights from comparative oncology. Effective implementation of One Health strategies requires coordinated governance that bridges research, policy, and clinical practice, ensuring that scientific advances translate into meaningful public health outcomes. For clinicians, adopting a One Health perspective implies expanding their role beyond individual patient care to include active participation in prevention, interdisciplinary collaboration, and public health advocacy. This includes promoting lifestyle modifications, recognizing environmental and occupational risk factors, supporting vaccination and screening programs, and contributing to the translation of One Health evidence into clinical practice and public health action. Future research should focus on developing integrative methodologies, including cross-species surveillance, exposure modelling, and translational epidemiology, to better understand complex carcinogenic pathways. Through transdisciplinary collaboration, this approach has the potential to strengthen cancer prevention, control, and management while developing evidence-based strategies that simultaneously protect human, animal, and environmental health.

## Figures and Tables

**Table 1:** Global Cancer Profile by Human Development Index Classification.

Countries by HDI *	Estimated New Cancer Cases 2022 [24]	Mortality 2022 [24]	Projected New Cancer Cases 2050 (Variation in %) [25]	Most Common Cancer Types [26]	Main Risk Factors
Very High HDI	9,296,171 (46.6%)	3,643,502 (37.4%)	13,168,937 (+41.7%)	Lung (13.6%), Breast (10.9%), Colorectum (10.4%), Prostate (7.8%), Stomach (5.0%), Thyroid (4.6%)	High body mass index, physical inactivity, unhealthy diets, smoking, alcohol consumption, reproductive behaviors [27,28,29,30]
High HDI	7,436,122 (37.2%)	3,991,272 (41.0%)	12,199,088 (+64.1%)		
Medium HDI	2,424,245 (12.1%)	1,560,054 (16.0%)	4,829,078 (+99.2%)	Breast (14.4%), Cervix uteri (8.9%), Lung (6.1%), Lip and Oral Cavity (6.1%), Colorectum (5.8%)	Infectious agents (human papillomavirus, hepatitis B and C virus, *Helicobacter pylori*, *Schistosoma haematobium*, *Opisthorchis viverrine*, *Clonorchis sinensis*) [30,31,32], smoking, alcohol consumption, diet [30,33,34,35]
Low HDI	812,211 (4.1%)	544,600 (5.6%)	1,966,488 (+142.1%)		

* HDI—Human Development Index.

**Table 2:** Animal models in comparative oncology: representative cancer types and their translational relevance to human cancer research.

Animal Model	Cancer Type	Relevance to Translational Research
Rodent [166,167]	Breast, Lung, Colorectal, Melanoma	Genetically engineered models, rapid tumor progression, widely used in drug screening, cost-effective model.
Dog [168,169,170,171,172]	Osteosarcoma, Hemangiosarcoma, Mammary Tumors, Bladder Cancer, Gliomas, Melanoma, Lymphoma	Similar spontaneous tumors to humans, excellent model for immunotherapy and metastasis studies.
Cat [173,174]	Oral Squamous Cell Carcinoma, Lymphoma, Mammary Tumors	Comparable tumor behavior to humans, useful for studying naturally occurring cancers.
Zebrafish [175]	Melanoma, Brain Tumors, Leukemia	High-throughput screening, developmental cancer studies, transparent embryos allow real-time tumor monitoring.

## Data Availability

No new data were created or analyzed in this study.

## Abbreviations

The following abbreviations are used in this manuscript:

BMI	Body Mass Index
CDC	Centers for Disease Control and Prevention
COVID-19	Coronavirus Disease 2019
DNA	Deoxyribonucleic Acid
ESMO	European Society for Medical Oncology
FAO	Food and Agriculture Organization of the United Nations
GDP	Gross Domestic Product
HBV	Hepatitis B Virus
HCV	Hepatitis C Virus
HDI	Human Development Index
HPV	Human Papillomavirus
IARC	International Agency for Research on Cancer
INT $	International dollars at constant 2017 prices
MeSH	Medical Subject Headings
NCD	Non-communicable Diseases
OHHLEP	One Health High-Level Expert Panel
PM	Particulate Matter
PM_2.5_	Fine Particulate Matter
PM_10_	Particulate Matter with an aerodynamic diameter ≥ 10 µg
RNA	Ribonucleic Acid
SDI	Social Development Index
TBL	Tracheal, Bronchus, and Lung
UN	United Nations
UNDP	United Nations Development Program
UNEP	United Nations Environment Program
UNICEF	United Nations International Children’s Emergency Fund
UNSIC	United Nations System Influenza Coordination
UV	Ultraviolet
UK	United Kingdom
USA	United States of America
WHO	World Health Organization
WOAH	World Organization for Animal Health
YLLs	Years of Life Lost
